# Predicting potential drug-drug interactions on topological and semantic similarity features using statistical learning

**DOI:** 10.1371/journal.pone.0196865

**Published:** 2018-05-08

**Authors:** Andrej Kastrin, Polonca Ferk, Brane Leskošek

**Affiliations:** Institute of Biostatistics and Medical Informatics, Faculty of Medicine, University of Ljubljana, Ljubljana, Slovenia; National Chiao Tung University College of Biological Science and Technology, TAIWAN

## Abstract

Drug-drug interaction (DDI) is a change in the effect of a drug when patient takes another drug. Characterizing DDIs is extremely important to avoid potential adverse drug reactions. We represent DDIs as a complex network in which nodes refer to drugs and links refer to their potential interactions. Recently, the problem of link prediction has attracted much consideration in scientific community. We represent the process of link prediction as a binary classification task on networks of potential DDIs. We use link prediction techniques for predicting unknown interactions between drugs in five arbitrary chosen large-scale DDI databases, namely DrugBank, KEGG, NDF-RT, SemMedDB, and Twosides. We estimated the performance of link prediction using a series of experiments on DDI networks. We performed link prediction using unsupervised and supervised approach including classification tree, *k*-nearest neighbors, support vector machine, random forest, and gradient boosting machine classifiers based on topological and semantic similarity features. Supervised approach clearly outperforms unsupervised approach. The Twosides network gained the best prediction performance regarding the area under the precision-recall curve (0.93 for both random forests and gradient boosting machine). The applied methodology can be used as a tool to help researchers to identify potential DDIs. The supervised link prediction approach proved to be promising for potential DDIs prediction and may facilitate the identification of potential DDIs in clinical research.

## Introduction

Combined use of multiple drugs at the same time (i.e., polypharmacy) is common in modern pharmacotherapy [1], particularly in older population who has required continuous treatment for one or more chronic diseases [2]. Empirical evidence reported that the percentage of the U.S. population taking three or more drugs increased for 12% in years 1988–1994 to 21% in years 2007–2010 [3]. In such settings drugs may interact; they are not independent from one another. Drug-drug interaction (DDI) is an event in which one drug influences the pharmacologic effect of another drug when both are administered together [4, 5]. Identifying DDIs is a critical process in drug industry and clinical patient care, especially in drug administration [6].

Adverse drug reactions (ADRs) are harmful reactions that are caused by intake of medications [7]. Many ADRs are not identified during clinical studies (i.e., before a drug is approved by a government). Liu [8] recently demonstrated that about 10% of all possible drug pairs may probably induce ADRs through DDIs. Therefore, one of the fundamental aspects in pharmacovigilance—a research field related to the detection and prevention of ADRs [9]—is to generate new knowledge about DDIs. Despite several resources for DDIs [10] (e.g., DrugBank, Drugs.com), a study has demonstrated that none of the actual public databases provide a tolerable coverage of all the known DDIs; these databases are either incomplete or they record a large number of irrelevant interactions [11]. Additionally, the great majority of DDIs is hidden in a crowd of unstructured textual data which is expanding at a large scale [12]. For example, as of date of this writing simple PubMed search returns about 150000 bibliographic citations which include MeSH term ‘Drug Interaction’. Hence, the main motivation behind this study is consideration of computerized approach to identify potential DDIs.

DDIs may be naturally represented as a network in which nodes refer to different drugs and relationships between them designate their interactions [13, 14]. Complex networks fascinate many researchers after the small-world [15] and scale-free [16] features were recognized in numerous real-life networks, such as the Web and large social networks that capture relationships between actors. The network induced can be employed to elucidate the architecture and dynamics of a complex system and assist us in identification of relevant topological properties, interesting patterns, and predicting future trends. Various studies have already been performed in pharmacology with interesting applications of complex networks, including DDIs prediction (e.g., [17, 18]). There are three main benefits of processing DDIs with network analysis approach [19]: (i) researcher can predict potential, previously unknown, DDIs; (ii) certain (insignificant) DDIs will be avoided in such knowledge representation; and (iii) relationships which link pharmacodynamic and pharmacokinetic drug characteristics to DDIs can be explored.

A plethora of statistical methods were employed and developed to predict DDIs. An extensive overview of recent approaches is presented separately in the next section. Existing methods may be categorized into three main approaches to DDI prediction: (i) a similarity-based approach, (ii) classification-based approach, and (iii) text mining approach. A similarity-based methods are based on the assumption that similar drugs may interact with the same drug. For instance, two drugs may interact if they have similar molecular profile. Classification-based techniques mimic the DDI prediction task as a binary classification problem. For example, drug-drug pairs are represented as feature vectors, while target variable is represented by presence or absence of interactions. A particular instance of classification-based methods is link prediction, which aim is to assess the probability that a relation exists between pair of nodes in a network, based on observation of topology of existing nodes and their attributes [20, 21]. Finally, text-mining methods employ natural language processing techniques to extract plausible relations among drugs from unstructured data sources (e.g., from MEDLINE citations). However, Abdelaziz et al. [22] identified several issues that are overlooked by a great majority of DDI prediction studies: (i) inability to predict newly developed drugs, (ii) failure to handle extreme data skewness of DDI pairs, (iii) relying the analysis only on selected data sources (mainly DrugBank), and (iv) careless evaluation techniques which is reflected by employing area under the ROC curve as the main evaluation metric to assess the quality of prediction. All these limitations encourage us to perform a new, improved experiment.

In this study we examine link prediction from the viewpoint of predicting potential DDIs. The main objectives of this work are: (i) to represent the process of discovering potential DDIs as a binary classification task in which features are represented as topological and semantic measures between drugs, and (ii) to evaluate performance of unsupervised and supervised machine learning methods for predicting potential DDIs. This study is different from other related studies in the following facets: (i) we use broader set of databases for DDIs prediction including DrugBank, KEGG, NDF-RT, SemMedDB, and Twosides; (ii) besides network-based features we also include semantic-based features, for instance chemical information of a drug and assigned Medical Subject Headings (MeSH); (iii) regarding methodological considerations we assume balanced distribution of DDI pairs; (iv) in addition to unsupervised approach we also include supervised statistical learning methods; and (v) last but not least, the study relies on comprehensive statistical evaluation and on manual evaluation performed by trained pharmacist.

## Related work

A recent comprehensive review of DDI detection utilizing clinical resources, scientific literature, and social media is given by Vilar et al. [23]. In previous section we defined three approaches to DDIs prediction, namely similarity-based approach, classification-based approach, and text mining approach. We review the most recent literature for each of the approaches in the next paragraphs.

The similarity-based approach exploits the idea of biological profiles which are used to compare drugs and infer new molecular properties [24]. Gottlieb et al. [25] performed statistical validation by considering various types of drug-drug similarities, including chemical-based and side-effect-based similarity. Vilar et al. [26] developed new approach appropriate for large scale data that detects DDIs based on similarity of molecular structural properties. Li et al. [27] presented a Bayesian network model which was combined with a similarity algorithm to predict the drug pairs from drug molecular and pharmacological features. Zhang et al. [28] developed an integrative label propagation framework to model DDIs by integration of ADRs and chemical structures. Sridhar et al. [29] developed a probabilistic approach for predicting DDIs. They used probabilistic soft logic framework which is highly scalable. The evaluation demonstrated of more than 50% improvement over baselines. Ferdousi et al. [30] reported on a methodology for DDIs modeling based on comparison of functional profiles of drugs, where drug profiles were constructed using carriers, transporters, enzymes, and targets information. They predicted over 250000 potential interactions. Takeda et al. [31] predicted DDIs based on structural similarities and the interaction networks that consist of pharmacokinetics and pharmacodynamics properties.

Classification-based approaches mimic the prediction of DDIs as a two-class classification task. Cami et al. [32] defined DDIs as combinations of feature vectors and then employ logistic regression model to predict future interactions. Their model achieves a sensitivity of 48% with a specificity of 90%. Cheng and Zhao [33] used four DDI similarity measures and applied various statistical learning methods (naive Bayes, classification tree, *k*-nearest neighbors, logistic regression, and support vector machine) to learn interactions between pairs of drugs. Jamal et al. [34] studied neurological ADRs. They use various properties of drugs including biological, chemical, phenotypic, and their combinations. They used feature selection based on relief to detect most important variables and then employed advanced statistical techniques to predict side effects. Abdelaziz et al. [22] developed a large-scale similarity-based framework that predicts DDIs using link prediction. The system can predict both novel DDIs among existing drugs as well as newly developed drugs. Similarly, Lu et al. [35] studied whether classical similarity measures provide plausible approach to drug-target interaction prediction, when only information from network topology is available. They compare their method against restricted Boltzmann machines and demonstrated higher precision of the proposed approach. Zhang et al. [18] collected a variety of information sources (i.e., data about substructures, targets, enzymes, transporters, pathways) and build prediction models using neighbor recommender, random walk, and matrix perturbation method. They demonstrated that the methods based on ensemble learning could derive higher prediction performance than individual algorithms. Hameed et al. [36] developed a methodology for DDI prediction that is especially useable in situations when true negative instances for training are inadequate.

Information about DDIs in the research literature is increasing rapidly. Third line of research thus utilizes text mining methods to infer novel DDIs. Duke et al. [37] perform literature discovery approach on large health information exchange data repository to predict and evaluate new DDIs. Their method could identify new clinically significant DDIs and also supports mining for their potential biological roots. Huang et al. [38] presented a method that estimates the strength of network connection between drug targets to predict pharmacodynamic DDIs with 82% accuracy. Tari et al. [39] proposed a novel approach that integrates automated reasoning techniques and text mining do derive new enzyme-based DDIs from MEDLINE abstracts. Manual evaluation revealed about 81% accuracy of their approach. Gottlieb at al. [25] introduced an interaction prediction framework that allows the inference of both pharmacokinetic and pharmacodynamic DDIs. They reported high sensitivity and specificity rates of the proposed approach. Lu et al. [17] recently described an automatic approach for the description of the mechanism of interactions using MEDLINE MeSH descriptors. Authors reported high accuracy for identification of appropriate MeSH headings, including drugs and proteins. Besides scientific literature, social media also provides promising approach that can be useful in detection of DDIs [23]. For example, Hamed et al. [40] presented computational framework that detects DDI patterns from Twitter hashtag-based networks.

## Materials and methods

### Drug-drug interaction networks

We compiled knowledge networks by using DDI data from five public drug databases, including DrugBank, KEGG, NDF-RT, SemMedDB, and Twosides. We formed a pair of drugs if both are involved in one adverse DDI. DDIs are typically represented as directed connections. In this work the direction of the interaction was ignored.

#### DrugBank

DrugBank is an encyclopedic Web repository containing complete biochemical and pharmacological data about drugs, including biological mechanisms and targets information [41]. Most of the information in DrugBank is throughly curated from research literature. Currently, DrugBank lists 10376 drug entries and 577712 directed interactions among them. In this study we used version 5.0 of the DrugBank which was obtained from the DrugBank Web page (https://www.drugbank.ca) on August 1, 2017. We parsed the DDI information from the provided XML file and compiled an edgelist of drug identifiers combinations.

#### KEGG

KEGG (Kyoto Encyclopedia of Genes and Genomes) is one of the most complete biomedical sources consisting of metabolic pathways from various species. KEGG DRUG is an exhaustive compilation of approved drugs in Europe, USA and Japan unified based on chemical structures [42]. It contains rich information about chemical structures and additional data such as DDIs, target molecules and therapeutic categories. KEGG DRUG provides graphical representation of the groups of chemical structural patterns, therapeutic categories, their relationships, and the history of drug development. The version used in this study was downloaded from the KEGG FTP server (ftp://ftp.genome.jp/pub/kegg/medicus/drug/) on August 1, 2017. The KEGG DRUG database contains 10340 drug entries and 500254 directed interactions. Mapping to DrugBank identifiers results in 1194 unique compounds and 52609 directed interactions.

#### NDF-RT

The National Drug File Reference Terminology (NDF-RT) is a collection of drug interactions previously provided by the US Veteran’s Administration (VA) for use in VA care [43]. First we prepared list of NDF-RT raw interactions by utilizing National Center for Biomedical Ontology SPARQL service (http://sparql.bioontology.org/sparql). Query was executed on August 1, 2017. Raw database contains 10530 directed interactions among compounds. Next we mapped these interactions to DrugBank identifiers employing UMLS Metathesaurus and using the rxcui field as a mapping key. Postprocessed database finally contains 701 mapped DrugBank identifiers and 8044 interactions among them.

#### SemMedDB

SemMedDB is a database of semantic predications (i.e., subject-relation-object triples) parsed from MEDLINE bibliographic database abstracts by the SemRep tool [44]. Subject and object arguments of each predication correspond to concepts from the Unified Medical Language System (UMLS) Metathesaurus while relations coincide with links from the UMLS Semantic Network. SemMedDB contains information from about 91 million predications from all of the MEDLINE citations (approximately 27 million bibliographic records as of this writing). We used the version v.30 of the SemMedDB database in this study that processed the MEDLINE up to end of June 2017. In this study, all ‘INTERACTS_WITH’ relationships between pairs of drugs were used as potential DDIs. Preprocessed database contains 1447792 directed interactions among UMLS concepts that refer to drugs. Next we use MRCONSO table from UMLS Metathesaurus to map UMLS concepts to DrugBank identifiers. Final database of interactions contains 1688 compounds and 37287 interactions.

#### Twosides

Twosides is a comprehensive source of polypharmacy ADRs for combinations of drugs [45]. The version used in this study was obtained from the Twosides Web page on August 1, 2017. Interactions in Twosides database are restricted to only those that cannot be unambiguously ascribed to either drug alone. We parsed the interaction information from the downloaded text file (http://tatonettilab.org/) and build a database of drug identifier pairs for the interacting compounds. We use PubChem (https://pubchem.ncbi.nlm.nih.gov/) identifiers to map Twosides identifiers to DrugBank identifiers. Final database of interactions contains 340 unique compounds and 19020 interactions.

### Data representation

Consider an undirected and unweighted network which is depicted as a simple graph *G*(*V*, *E*) that consists of a set of nodes *V* referring to drugs and a set of edges *E* representing interactions between drugs. Let |.| represent the cardinality of the set. Let us first introduce some notation which is essential to understand the basics of the link prediction; for a comprehensive introduction to the technical details of link prediction we refer the reader to excellent reviews by Liben-Nowell and Kleinberg [20] or Lü and Zhou [21].

Let *U* be the universal set containing (|*V*| ⋅ |*V*| − 1)/2 possible edges. By *U* − *E* we denote a set of non-existing links (or links that will appear later in time). The problem of link prediction is to predict these missing links. To test prediction algorithms we split the set of observed links *E* into two partitions: the training partition *E*^*T*^ and test partition *E*^*P*^. It follows that *E*^*T*^ ∪ *E*^*P*^ = *E* and *E*^*T*^ ∩ *E*^*P*^ = ⌀. In this study, we split each data set *E* into 66% training and 33% test data. For all pairs of nodes in the training data we calculate similarity measure, which reflects the chance that a pair of nodes will interact in the test data set. In terms of machine learning, each pair of nodes serve as a positive or negative example, depending on whether those node pairs form a link in the test network. We organize the whole network as a list of relations

$$U=\{\left\langle u_{1},u_{2}\right\rangle ,\left\langle u_{1},u_{3}\right\rangle ,\ldots ,\left\langle u_{i},u_{j}\right\rangle ,\ldots ,\left\langle u_{n-1},u_{n}\right\rangle \},$$

where *n* is the number of nodes in the network. Each term of the list comprises a feature vector and a relationship (i.e., class) label. The label is 1 when *u*_*i*_ following *u*_*j*_ and 0 otherwise. A feature vector is composed by the two feature subsets, as described in the next section.

Our basic assumption is that similar nodes more probably form a potential DDI. For each non-existent pair (*x*, *y*) in a test data, a link prediction algorithm provides a score *s*(*x*, *y*) ∈ *U* − *E*^*T*^ that is an estimate of the existence of link between nodes *x* and *y*.

### Feature extraction

Extracting a relevant set of features is one of the most critical part of any statistical learning algorithm. Traditional link prediction research considers mostly the topological features. In this study we augment the set of topological features with four semantic features.

#### Topological features

*Common neighbor (CN)*. Due to its simplicity this is one of the most commonly used measure in link prediction [46]. For a node *x*, let Γ(*x*) denotes a set of neighbors of *x*. For nodes *x* and *y* the CN is defined as the number of nodes that *x* and *y* have in common. CN gives the relative similarity between a pair of nodes. CN is formally defined as

$$s_{x,y}^{CN}=|\Lambda_{x,y}|=|\Gamma \left(x\right)\cap \Gamma \left(y\right)|.$$

*Jaccard’s coefficient (JC)*. It is a normalized version of CN. JC assumes higher values of node pairs (*x*, *y*), which have many common neighbors proportionate to the total number of neighbors they have [47]. JC is formally defined as

$$s_{x,y}^{JC}=\frac{\left|\Gamma (x)\cap \Gamma (y)\right|}{\left|\Gamma (x)\cup \Gamma (y)\right|}.$$

*Adamic/Adar index (AAI)*. This index was first proposed for measuring similarity between two Web pages [48]. AAI definition is related to JC, with a correction that lower-connected neighbors are weighted more heavily. AAI is formally defined as

$$s_{x,y}^{AAI}=\sum_{z\in \Gamma (x)\cap \Gamma (y)}\frac{1}{\text{log}|\Gamma (z)|}.$$

*Preferential attachment (PA)*. This is simply the product of the degrees of nodes *x* and *y*. This measure rest on an assumption that new edges more probably connect to higher-degree nodes than to lower-degree ones [49]. PA is defined as

$$s_{x,y}^{PA}=\left|\Gamma \left(x\right)\times \Gamma \left(y\right)\right|.$$

*Resource allocation (RAI)*. It is similar to AAI but it penalizes the common neighbors with higher degree more rigorously. RAI is formally defined as

$$s_{x,y}^{RAI}=\sum_{z\in \Gamma (x)\cap \Gamma (y)}\frac{1}{\left|\Gamma (z)\right|}.$$

*Common neighbors 1 (CCN)*. This measure begins with the base score given by |Λ_*x*,*y*_| and then for every neighbor *i* shared by *x* and *y*, CCN receives an additional point for every community that *x*, *b*, and *i* are all in. Formally, CCN is calculated as

$$s_{x,y}^{CCN}=|\Lambda_{x,y}|+\sum_{i\in \Gamma (x,y)}\left|C\left(i\right)\cap C\left(x\right)\cap C\left(y\right)\right|,$$

where *C*(*n*) is the set of node communities to which node *n* belongs.

*Resource allocation 1 (CRA)*. It is similar to the original resource allocation definition, but it gives extra weight to shared neighbors *i* that are in at least one community with both *x* and *y*, and weight *i*’s contribution toward the total score by the number of communities that *i* shares with *x* and *y*. CRA is formally defined as

$$s_{x,y}^{CRA}=\sum_{i\in \Gamma (x,y)}\frac{1+|C(i)\cap C(x)\cap C(y)|}{d(i)},$$

where *d*(*i*) is degree of node *i*.

*Within-inter cluster (WIC)*. WIC predicts link between a pair of nodes using information from within-cluster (W) and inter-cluster (IC) common neighbors of these nodes. A community detection must be performed on the network before applying this metric. Each vertex belongs to only one community. WIC is formally defined as

$$s_{x,y}^{WIC}=\frac{|\Lambda_{x,y}^{W}|}{|\Lambda_{x,y}^{IC}|+\delta },$$

where *δ* ≈ 0 is a small constant to prevent division by zero when $\Lambda_{x,y}^{W}=\Lambda_{x,y}$ causing $\Lambda_{x,y}^{IC}=⌀$.

#### Semantic features

*Drug therapeutic-based similarity (ATC)*. This type of similarity was evaluated through ATC codes. ATC coding system partitions compounds into different clusters according to the biological system or organ on which they act. The first level of the code which was used in this study indicates the anatomical main group. There are 14 main clusters (e.g., A—alimentary tract and metabolism, B—blood and blood forming organs). The ATC codes for all compounds were extracted from the main DrugBank file. There are 3322 unique ATC codes as of this writing in the DrugBank database. Each compound was represented by a binary vector in which elements refer to the presence or absence of the ATC codes. We used inverse document frequency (IDF) to discount ‘popular’ ATC codes following the formula

$$\text{IDF}\left(t,D\right)=\text{log}\frac{\left|D\right|}{n_{t}},$$

where *D* is the set of compounds and *n*_*t*_ is the number of compounds where the ATC code *t* appears. Drug therapeutic similarity of a pair of drugs is the cosine similarity of the corresponding IDF-weighted vectors.

*Chemical structure-based drug similarity (CHEM)*. First we represent a given set of compounds as SMILES (simplified molecular-input line-entry system) strings which were extracted from DrugBank. The SMILES strings were then converted into molecular extended fingerprints (1024 bits) using the R’s rcdk package. Finally, we converted a set of fingerprints into a Tanimoto similarity matrix using the fp.sim.matrix() function from rcdk package. The Tanimoto similarity coefficient [50] between compounds *x* and *y* is computed using the formula

$$s_{x,y}=\frac{c}{a+b-c},$$

where for bit-strings of length *n*, we suppose that *a* bits are set in the string for a compound *x*, *b* bits are set for a comparison compound *y*, and *c* bits are common to both strings. Values contained in the matrix represent the chemical structure similarity between each possible pair of compounds.

*MeSH-based similarity (MESH)*. MeSH is a controlled vocabulary which is used to index MEDLINE database. MeSH-based similarity is based on MeSH terms that are associated with DrugBank entries. There are 2072 different MeSH terms in the DrugBank database. As in the case of drug therapeutic-based similarity, each compound was represented by a binary vector whose elements represent the presence of the MeSH terms. The MeSH-based similarity is defined as the cosine similarity between the IDF-weighted MeSH vectors of the two corresponding compounds.

*Adverse drug effect-based similarity (ADE)*. For this type of similarity we use information provided by SIDER side effects database of drugs. SIDER provides data on marketed drugs and their known ADRs. The version used in this study (4.1) was obtained from the SIDER Web page [51]. There are 1430 drugs and 5868 side effects in the database. Each compound was represented by a vector with binary values in which elements represent the presence of the side effect terms. The side effect similarity of two compounds is defined as a cosine similarity between the IDF-weighted side effect vectors of the two compounds.

### Statistical learning

In this study we used unsupervised and supervised learning. Later was performed by using five state-of-the-art classifiers, namely classification tree (DT), *k*-nearest neighbors (*k*NN), support vector machine (SVM), random forest (RF) and stochastic gradient boosting also known as gradient boosting machine (GBM). These classifiers have become mainstream in modern statistical learning. A comprehensive overview of all learning methods is not in scope of this paper. However, in the following lines we will shortly introduce the basic background. For more deep insight please see Friedman et al. [52].

#### Unsupervised classification

For unsupervised classification we use combined similarity measure which is derived from standardized similarity scores for pairs of nodes based on topological and semantic properties of the networks. More formally, we define combined similarity measure as

$$s_{x,y}^{Comb}=\text{Avg}(s_{x,y}^{CN},s_{x,y}^{JC},\ldots ,s_{x,y}^{ADE}),$$

where Avg is arithmetic mean. A pair of drugs is predicted to have a link if its score is over a certain threshold *t*. Clearly, a lower threshold predicts more pairs to be links. In our settings we use *t* = 90th percentile as a threshold. For example, value of combined similarity above chosen threshold therefore predicts a link between selected nodes. We use class information as described previously in ‘Data representation’ section.

#### Classification tree

DT is built by partitioning instances into local subsets using a series of recursive splits. Each node of a tree is constructed by a logical rule, where instances below a certain threshold fall into one of the two child nodes, and instances above fall into the other child node. Partitioning continues until a terminal node, where data instances are assigned a class label [52]. The prediction for an instance is obtained by a majority vote of the instances reaching the same terminal node. Classifier was constructed using the rpart package in R.

#### *k*-nearest neighbors

*k*NN classifier defines the class of a test instance according on the majority vote of its *k* nearest neighbors from training data [52]. We set the value of *k* using internal 5-fold cross-validation. We used the Euclidean metric for calculating distances between data points. *k*NN classifier was implemented using the class package in R.

#### Support vector machine

SVM classifier maps the input data set into a high-dimensional feature space and then constructs a hyperplane to separate classes based on a maximum margin principle. We can choose various kernel functions including linear or nonlinear [52]. SVM classifier was implemented using the e1071 package in R. The penalty parameter was determined by an internal 5-fold cross-validation. Our implementation uses the linear kernel.

#### Random forest

RF is a statistical learning methodology that perform ensemble learning for classification. Ensemble consists of multiple classification trees [53]. We used bootstrap sampling on training data to grow each tree. We split each node using the best among a randomly selected subset of given features. Next, we combined class labels predicted by each tree in the forest. Majority vote is finally used to create final prediction. RF classifier was implemented using the ranger package in R.

#### Gradient boosting machine

GBM also provides ensemble learning, but the base learners in a GBM are weak learners [54]. The trees in GBM are not grown to the maximum possible extent as in RF. The GBM starts with an imperfect model (i.e., the base learner that is not grown maximally) and generates a new model by successively fitting the residuals of the current model, using the same class of base learners as the initial imperfect model. GBM classifier was implemented using the gbm package in R.

### Evaluation metrics

To estimate the quality of the proposed methodology, we performed two types of analyses: we performed statistical validation on selected DDI data sets as well as qualitative validation on a small subset of DDIs.

The performance of algorithms was evaluated by employing train-test schema. First we used ovun.sample() function from the ROSE package in R to create a representative sample of DDI pairs for each network. Models were trained and tuned using the caret package in R utilizing doMC package for parallel processing. We used createDataPartition() function to split the entire data set into training subset containing 66% of examples and a test subset containing 33% of examples. Model selection was carried out using 10-fold cross-validation on training subset, which is known to give the lowest bias and variance [52]. The model with the highest accuracy was selected as the candidate model and used to predict interactions in the testing dataset.

To benchmark the performance of our algorithms we used standard evaluation measures from statistical learning including precision, recall, *F*_1_ measure, area under the receiver operating characteristic (ROC) curve (AUROC), and area under the precision-recall curve (AUPR). Precision refers to the proportion of instances classified as positive that are actually positives, while recall refers to the proportion of true positive instances correctly classified as positives. *F*_1_ measure is used to integrate precision and recall into a single measure. ROC curve is a plot of true positive rate (sensitivity) vs. false positive rate (1—specificity). Despite its popularity, the ROC curve has some drawbacks including the inappropriateness for imbalanced data [55]. For this reason we also used the AUPR.

To evaluate statistically significant differences between classifiers across different networks, we followed the methodology proposed by Demšar [56] as implemented in scmamp package. We used Friedman test, which is a non-parametric alternative of repeated ANOVA design. The test is based on rank comparison that identify an overall effect of the choice of classifier on performance across multiple experiments. The null hypothesis is that all classifiers are equivalent. When the null hypothesis of the Friedman test is rejected (*p* < 0.05), we proceed with the Nemeny post-hoc test, which compares classifiers to each other across datasets and finds the statistical significance of differences between their average performance ranks. Lower ranks indicate superior performance but only differences over a certain critical difference are considered statistically significant.

### Software

We used custom AWK and Python scripts for data preprocessing. Similarity measures were implemented using NetworkX package in Python. Other numerical computations, including statistical learning were performed using R programming language for statistical computing and graphics. Complete programming code to reproduce the results of this study is accessible in GitHub repository at URL https://github.com/akastrin/ddi-prediction.

## Results

### Network characteristics

We first examine elementary statistical characteristics of the included DDI networks. The summary of the topological properties is presented in Table 1. The networks exhibit short average path length; in other words there are only about *L* = 2.47 hops on average from the node *x* to node *y* in the network. The average clustering coefficient of the networks is *C* = 0.46. Median diameter across the networks is six hops. On average, the giant component comprises practically all nodes.

**Table 1:** Basic characteristics of DDI networks.

Network	|*V*|	|*E*|	*c*	*D*	*L*	*C*	*GC*
DrugBank	2551	577712	452.93	6	2.27	0.52	1.00
KEGG	1194	52609	88.12	7	2.51	0.37	1.00
NDF-RT	701	8044	22.95	8	3.30	0.16	0.99
SemMedDB	1688	37287	44.18	6	2.58	0.44	1.00
Twosides	340	19020	111.88	3	1.68	0.83	1.00

*Legend:* |*V*|—number of nodes, |*E*|—number of edges, *c*—average degree, *D*—diameter, *L*—average path length, *C*—clustering coefficient, *GC*—size of giant component.

Next, we summarized the number of common edges between pairs of networks (Table 2). The proportion of intersections is defined as the number of overlapping edges divided by the smaller number of edges in each of the networks in pair. Results demonstrate that, though some duplicated drug-drug pairs exist, most of the pairs have low overlap proportions. This indicates that presented DDI networks are complementary to each other.

**Table 2:** Overlaps between data sources.

**Network**	**DrugBank**	**KEGG**	**NDF-RT**	**SemMedDB**	**Twosides**
DrugBank	296656	0.36	0.45	0.30	0.43
KEGG	11961	33474	0.14	0.04	0.04
NDF-RT	1790	576	4010	0.10	0.05
SemMedDB	8603	1077	390	28924	0.08
Twosides	7411	691	199	1396	17219

*Note:* The diagonal values represent the number of undirected edges in each network. The values in the lower triangle show the number of overlap between two networks. The values in the upper triangle refer to the proportions of overlap between the two networks. The proportion is defined as the number of intersections divided by the minimum number of the two networks.

### Performance evaluation

In this subsection we first delve into the results of unsupervised classification, and then we present performance evaluation of supervised classification.

The results of unsupervised classification are summarized in Table 3. Among all data sources Twosides network has the highest precision, but also the lowest recall. Overall, with the exception of DrugBank and Twosides, unsupervised classification shows low precision and high recall pattern. This results in high probability of false positives and a lower probability of misses. However, with the class imbalance data, recall of the positive class is often very low, which is not the case here. As suggested by the reviewer, we also report four other performance metrics (i.e., false negative rate, false positive rate, true negative rate, and true positive rate) that measure the classification efficiency on positive and negative classes independently (S1 Table).

**Table 3:** Unsupervised classification performances for link prediction on training and test data.

Network	Subset	Prec	Rec	F_1_	AUC	AUPR
DrugBank	train	0.63	0.68	0.65	0.93	0.70
	test	0.63	0.68	0.65	0.93	0.70
KEGG	train	0.28	0.63	0.38	0.91	0.32
	test	0.28	0.64	0.39	0.91	0.35
NDF-RF	train	0.09	0.58	0.15	0.83	0.10
	test	0.08	0.56	0.14	0.84	0.11
SemMedDB	train	0.16	0.80	0.27	0.93	0.45
	test	0.17	0.83	0.28	0.95	0.48
Twosides	train	0.96	0.30	0.46	0.90	0.83
	test	0.96	0.30	0.45	0.89	0.82

*Legend:* Prec—precision, Rec—recall, *F*_1_—*F*_1_ measure, AUC—area under the ROC curve, AUPR—area under the precision-recall curve.

Prediction performances for supervised learning are summarized in Tables 4 and 5, separately for training and test datasets. For training data, the Twosides network achieves the best performance (averaged across different classifiers) in terms of AUPR, followed by DrugBank, KEGG, SemMedDB, and NDF-RT. DrugBank and Twosides achieve high scores on both precision and recall, while other networks score much higher on precision than on recall. Other classification rates are presented separately in ‘Supplementary information’ (S2 Table).

**Table 4:** Classification performances for link prediction on training data.

Network	Classifier	Prec	Rec	F_1_	AUC	AUPR
DrugBank	DT	0.84	0.56	0.67	0.84	0.64
	*k*NN	0.86	0.70	0.77	0.98	0.89
	SVM	0.83	0.59	0.69	0.93	0.78
	RF	0.84	0.56	0.67	1.00	1.00
	GBM	0.83	0.65	0.73	0.96	0.82
KEGG	DT	0.68	0.34	0.46	0.78	0.42
	*k*NN	0.76	0.40	0.53	0.98	0.67
	SVM	0.70	0.21	0.33	0.79	0.45
	RF	0.68	0.34	0.46	1.00	1.00
	GBM	0.71	0.42	0.53	0.95	0.60
NDF-RF	DT	0.77	0.17	0.28	0.72	0.25
	*k*NN	0.59	0.05	0.10	0.98	0.37
	SVM	0.58	0.09	0.15	0.83	0.20
	RF	0.77	0.17	0.28	0.99	0.91
	GBM	0.91	0.28	0.43	0.94	0.53
SemMedDB	DT	0.76	0.24	0.36	0.75	0.36
	*k*NN	0.74	0.31	0.43	0.98	0.59
	SVM	0.70	0.27	0.39	0.87	0.48
	RF	0.76	0.24	0.36	0.99	0.97
	GBM	0.76	0.32	0.46	0.95	0.54
Twosides	DT	0.88	0.84	0.86	0.93	0.86
	*k*NN	0.91	0.81	0.86	0.97	0.95
	SVM	0.90	0.81	0.85	0.96	0.94
	RF	0.88	0.84	0.86	1.00	1.00
	GBM	0.92	0.87	0.89	0.98	0.96

*Legend:* Prec—precision, Rec—recall, *F*_1_—*F*_1_ measure, AUC—area under the ROC curve, AUPR—area under the precision-recall curve.

**Table 5:** Classification performances for link prediction on test data.

Network	Classifier	Prec	Rec	F_1_	AUC	AUPR
DrugBank	DT	0.83	0.55	0.66	0.84	0.63
	*k*NN	0.83	0.66	0.74	0.94	0.81
	SVM	0.83	0.58	0.69	0.93	0.78
	RF	0.83	0.55	0.66	0.98	0.92
	GBM	0.83	0.65	0.73	0.96	0.82
KEGG	DT	0.66	0.32	0.43	0.79	0.42
	*k*NN	0.68	0.35	0.46	0.88	0.51
	SVM	0.72	0.21	0.33	0.80	0.47
	RF	0.66	0.32	0.43	0.96	0.69
	GBM	0.67	0.37	0.48	0.95	0.55
NDF-RT	DT	0.60	0.12	0.20	0.70	0.20
	*k*NN	0.25	0.03	0.06	0.79	0.17
	SVM	0.56	0.07	0.13	0.87	0.21
	RF	0.60	0.12	0.20	0.91	0.36
	GBM	0.63	0.15	0.24	0.90	0.27
SemMedDB	DT	0.73	0.25	0.38	0.75	0.36
	*k*NN	0.68	0.30	0.42	0.86	0.45
	SVM	0.69	0.29	0.41	0.89	0.50
	RF	0.73	0.25	0.38	0.96	0.55
	GBM	0.68	0.31	0.43	0.96	0.53
Twosides	DT	0.83	0.82	0.82	0.90	0.80
	*k*NN	0.85	0.77	0.81	0.93	0.90
	SVM	0.86	0.80	0.83	0.95	0.92
	RF	0.83	0.82	0.82	0.96	0.93
	GBM	0.86	0.83	0.85	0.95	0.93

*Legend:* Prec—precision, Rec—recall, *F*_1_—*F*_1_ measure, AUC—area under the ROC curve, AUPR—area under the precision-recall curve.

To better understand the performance of classifiers, we evaluated the significance of their differences in AUPR. We applied Friedman test to compare the classifiers over multiple datasets. For training data the null hypothesis is rejected (*χ*^2^(4) = 18.4, *p* = 0.001). Therefore, significant differences exist in AUPR measure of the included classifiers.

**Fig 1:**
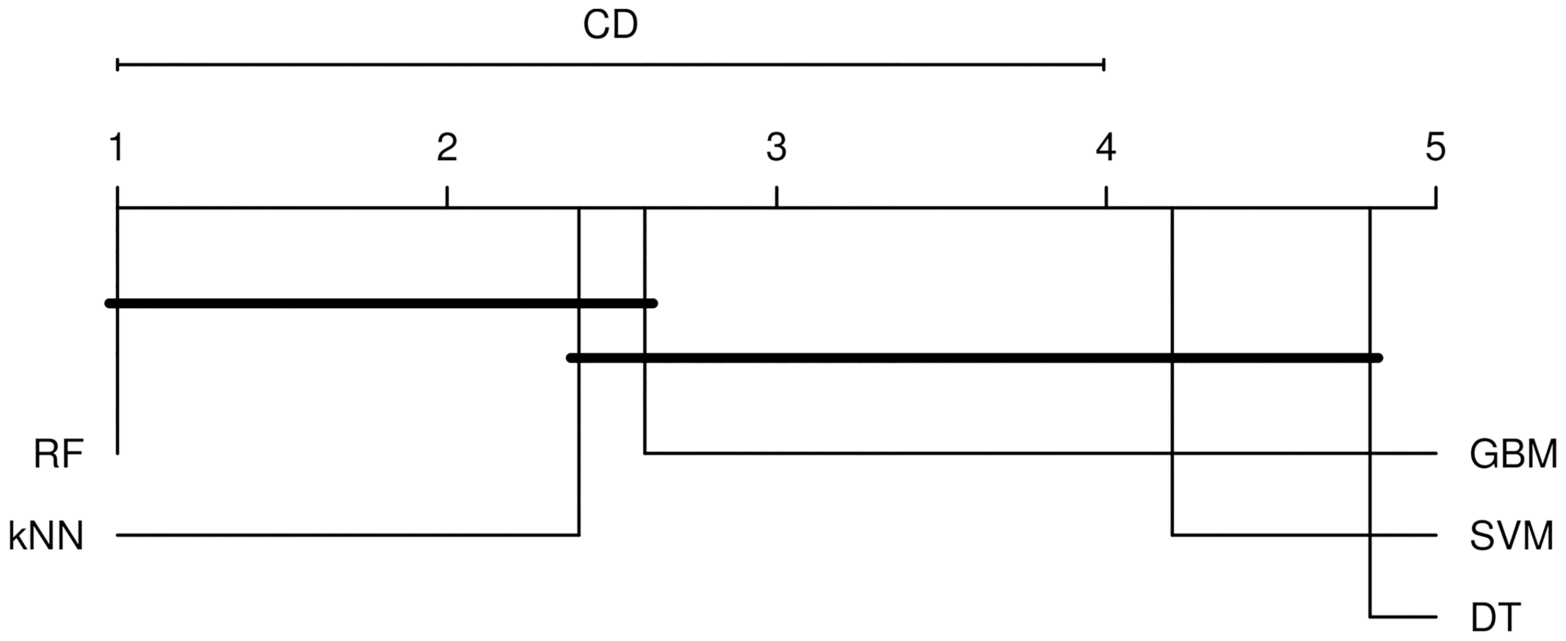
Critical difference (CD) plot for training data. Plot shows the pairwise differences in performance among classifiers. The horizontal scale shows the average rank of each classifier, with smaller ranks indicating better performance. Classifiers connected by a dark line had statistically identical performance at the *p* = 0.05 level.

After determining that an overall effect of classifier choice exists, we examined the pairwise differences among the classifiers using Nemenyi’s test, which is a post-hoc test based on studentized range distribution with a correction for multiple comparisons. Statistically significant differences exist for classifiers RF—DT (*p* = 0.001) and RF—SVM (*p* = 0.009). Fig 1 shows a critical difference (CD) diagram that depicts the average ranks of the classifiers for AUPR. We observe that RF, *k*NN, and GBM perform better than SVM and DT. When we also include unsupervised classifier into comparison as a baseline, the differences between Unsupervised—RF (*p* = 0.001) and Unsupervised–*k*NN (*p* = 0.034) emerge as statistically significant.

For test data (Table 5), the Twosides network achieves the best performance (averaged across different classifiers) in terms of AUPR, followed by DrugBank, KEGG, SemMedDB, and NDF-RT. In terms of precision and recall the pattern of values is similar to training regime in Table 4; DrugBank and Twosides score high on both precision and recall, while other networks exhibit much higher precision than recall. Other classification rates are presented separately in ‘Supplementary information’ (S3 Table).

**Fig 2:**
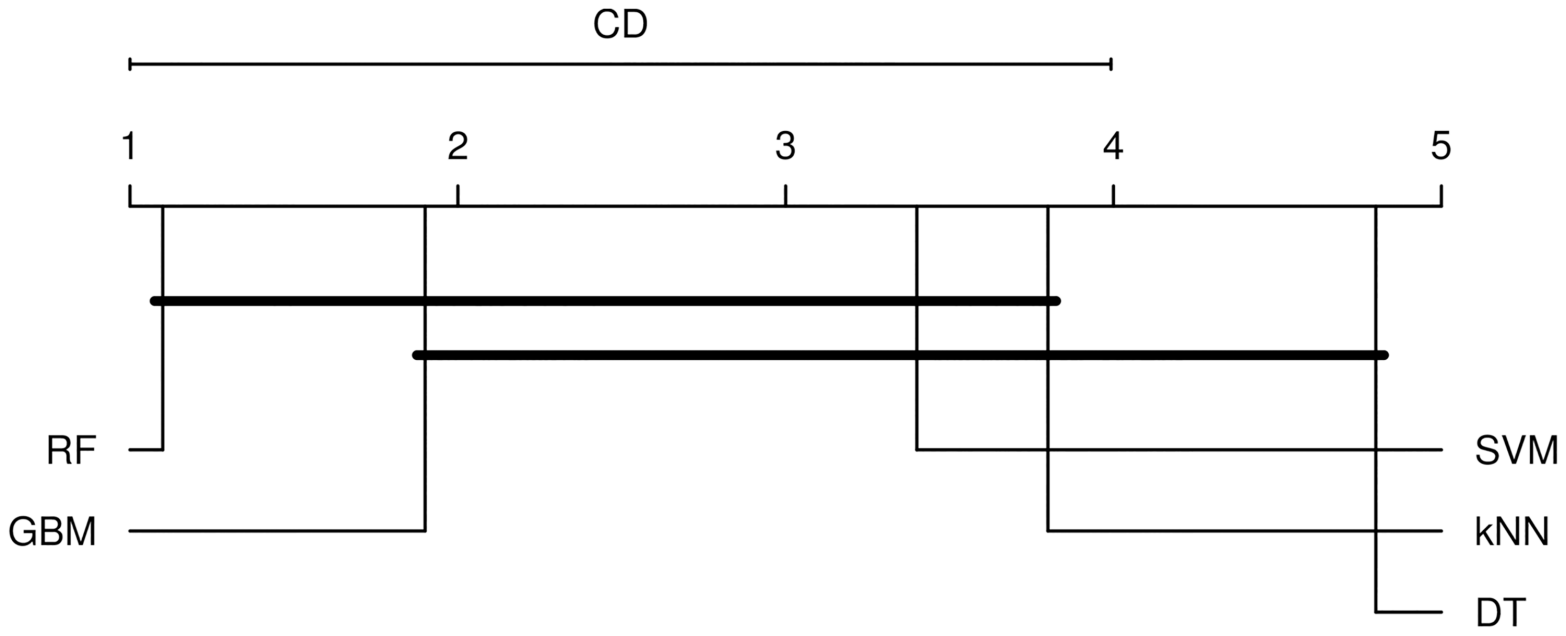
Critical difference (CD) plot for test data. Plot shows the pairwise differences in performance among classifiers. The horizontal scale shows the average rank of each classifier, with smaller ranks indicating better performance. Classifiers connected by a dark line had statistically identical performance at the *p* = 0.05 level.

For test data the Friedman test rejects the null hypothesis that all classifiers perform similarly (*χ*^2^(4) = 17.72, *p* = 0.001). Hence, we applied a post-hoc Nemenyi test to evaluate the significance of the differences in the ranks. Statistically significant differences exist between classifiers RF—DT (*p* < 0.001), RF–*k*NN (*p* = 0.033), and GBM—DT (0.010). We observe similar ranking in Fig 2 as with training data; ranks of RF, GBM, SVM, and *k*NN do not present a significant difference. DT classifier performs worse than the others. Finally, we included unsupervised classifier into comparison as a baseline classifier. The differences between Unsupervised—RF (*p* = 0.001) and Unsupervised—GBM (*p* = 0.010) show as statistically significant.

### Feature importance

**Fig 3:**
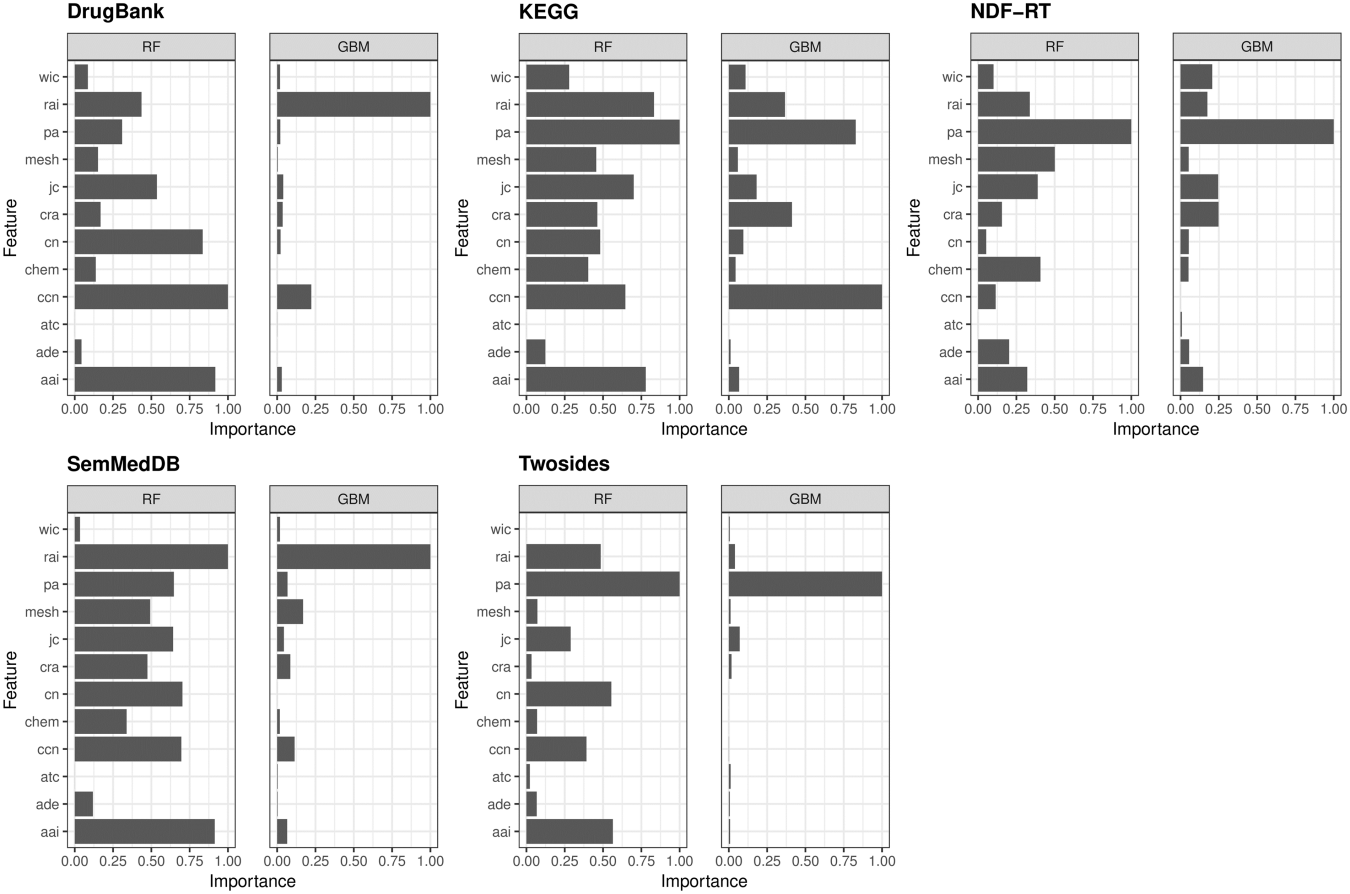
Relative feature importance. Normalized average relative feature importance for both learning methods (RF and GBM) for all included networks. For each model we quantified relative importance by a weight between 0 and 1 for each feature.

In addition to performance comparison, we analyzed the most important features that contribute to the statistical learning models. One of the nice features about RF and GB is that they provide indication of which features are most important in the classification. We quantified relative importance by assigning a score (0–100) for each feature as shown in Fig 3. The variable with the larger variable importance will have a value of 100 corresponding to the maximum variable importance and 0 will correspond to the lowest variable importance. We used MeanDecreaseGini measure which is based on Gini impurity index [52] as a metric for feature importance. However, it is beyond the scope of this paper to provide extensive details about the derivation of variable importance for both models. The interested reader can find details about this topic in appropriate literature [52]. Absolute importance scores for both classifiers are included in ‘Supplementary information’ file (S4 Table).

To summarize results in Fig 3, for RF pa is most important feature overall (*M* = 0.79), following by aai (*M* = 0.70). On the other side of scale are wic (*M* = 0.10) and atc (*M* = 0.00) with the lowest importance score. Among semantic features, the MeSH-based similarity feature has the best score (*M* = 0.33). The pattern is similar for GBM classifier with pa as most important feature (*M* = 0.58) and atc with the lowest importance (*M* = 0.00). MeSH-based similarity is again scored as the best semantic feature (*M* = 0.06). Results clearly show that topological information is more important than semantic information.

**Fig 4:**
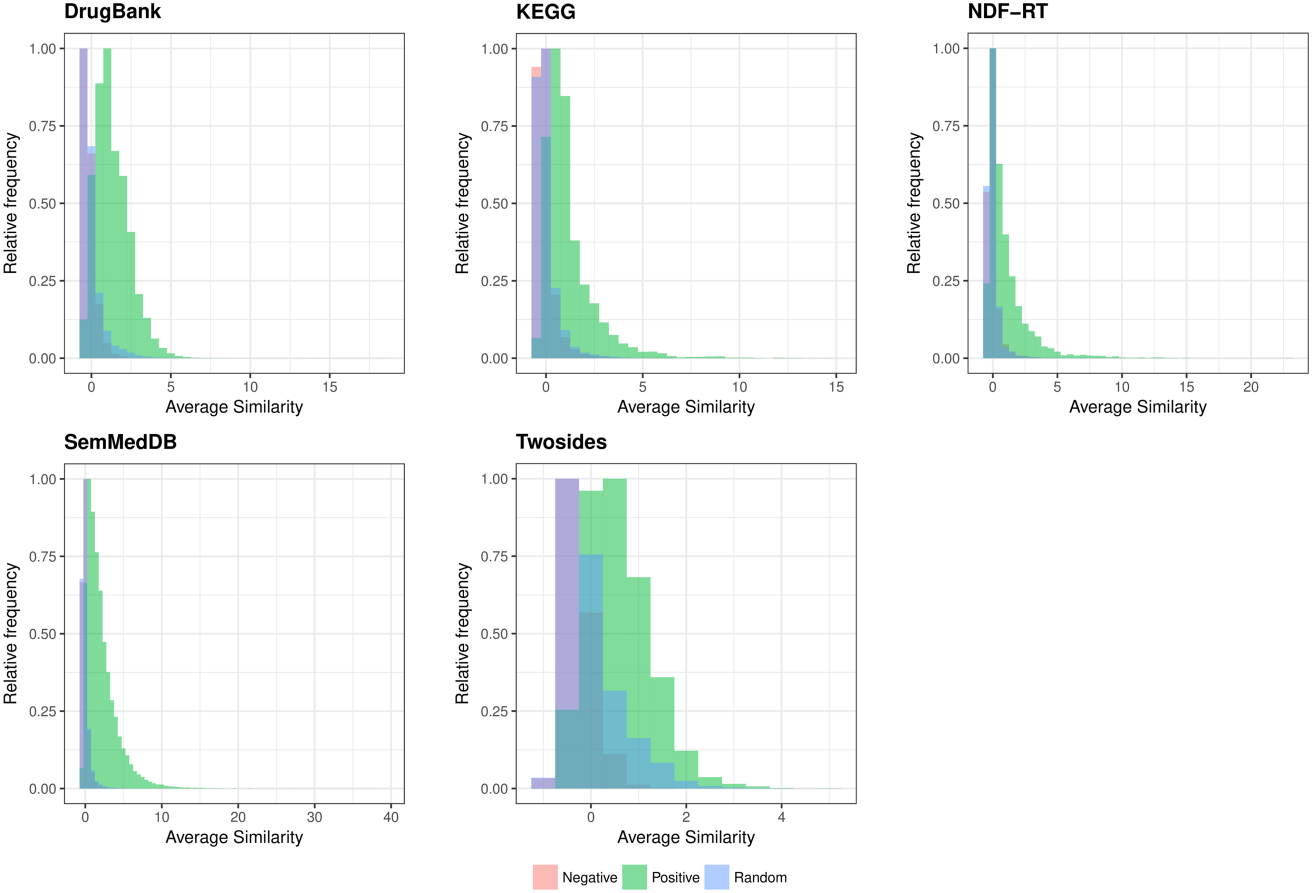
Distribution of average similarity in positive, negative, and random drug-drug pairs. Topological and semantic similarity measures were averaged across drug-drug pairs. All similarities were scaled before averaging.

Moreover, using the approach suggested in [33] we found that average similarity of positive DDI pairs is statistically significantly higher than those of negative drug pairs and random drug pairs for all five networks. Statistical significances were calculated using Kruskal-Wallis rank sum test; all values were *p* < 0.001. Differences between positive, negative, and random drug pairs are graphically depicted in Fig 4. This finding confirm our main hypothesis that similar compounds (i.e., measured by topological and semantic features) tend to have high potential of DDIs.

**Fig 5:**
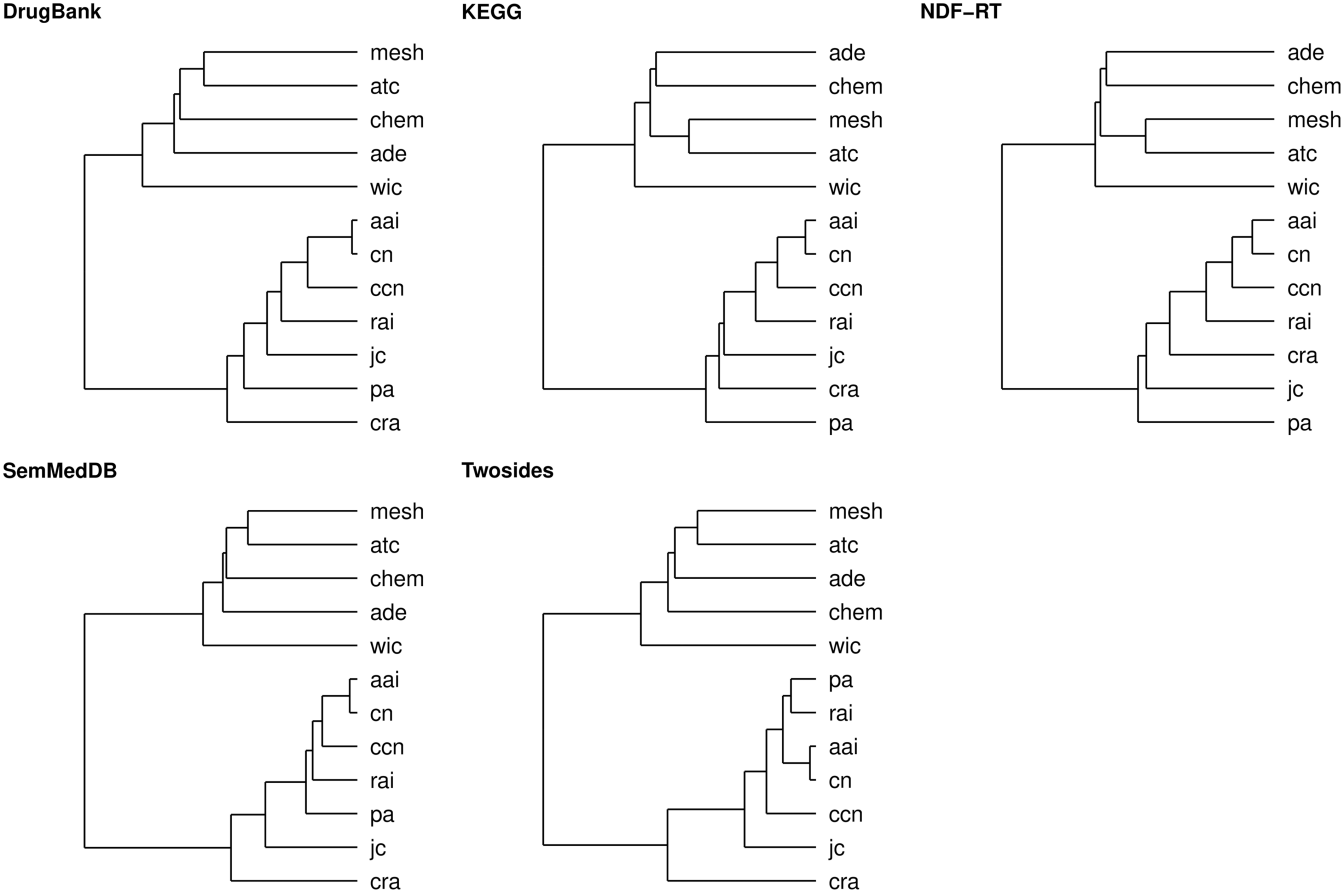
Hierarchical clustering of features. Hierarchical clustering was performed using Euclidean distance as a metric and using Ward method. All features were scaled before applying clustering.

Next, we also performed hierarchical clustering of data (i.e., we combined training and test data), to get insight into relationships between features which were used in statistical learning procedure. Clustering results are presented as series of dendrograms in Fig 5. The number of clusters across networks is constant; each data set could be partitioned into two clusters. First cluster is composed of features that reflect topological similarity between nodes: aai, ccn, cn, cra, jc, pa, and rai. Semantic similarity features (i.e., ade, atc, chem, and mesh) are grouped into the second cluster. The wic is somehow attached to the second cluster, although it may form separate cluster.

### Case study

In this subsection we study a list of top 15 predicted interactions between compounds based on DrugBank database (Table 6). We offer a reader a concise description of each compound as provided by the PubChem service and a possible explanation of an interaction. We also list a frequency of PubMed documents that contain both compound names in the title or abstract field. (Co-occurrence frequency was obtained through PubMed service using the query ‘compound_name_1[TW] AND compound_name_2[TW]’). This frequency could serve as an indicator of novelty for our discoveries. A trained pharmacist (PF) reviewed all interactions and suggests a most plausible interpretation for the cause of interaction. Below we present a subset of four interactions, which seem most interesting to us.

**Table 6:** Top 15 most plausbile novel interactions extracted from DrugBank database.

Compound 1		Compound 2		Freq
ID	Name	ID	Name	
DB13136	Fluindione	DB09268	Picosulfuric acid	0
DB12768	BCG vaccine	DB09268	Picosulfuric acid	0
DB05440	SRP 299	DB05322	INGN 201	0
DB00358	Mefloquine	DB01083	Orlistat	0
DB00657	Mecamylamine	DB09214	Dexketoprofen	0
DB01418	Acenocoumarol	DB12768	BCG vaccine	0
DB00358	Mefloquine	DB06148	Mianserin	12
DB00498	Phenindione	DB12768	BCG vaccine	0
DB01092	Ouabain	DB01396	Digitoxin	391
DB01032	Probenecid	DB12768	BCG vaccine	0
DB06148	Mianserin	DB01083	Orlistat	0
DB00495	Zidovudine	DB01223	Aminophylline	1
DB00196	Fluconazole	DB00176	Fluvoxamine	13
DB01323	St. John’s Wort	DB09280	Lumacaftor	0
DB01174	Phenobarbital	DB00289	Atomoxetine	2

Legend: ID—DrugBank database identifier, Name—Drugbank compound name, Freq—number of PubMed documents citing both compound names.

Fluindione (DB13136) is a vitamin K antagonist and is under investigation for the treatment of venous thrombosis, pulmonary embolism, permanent atrial fibrillation, and anticoagulation therapy. On the other hand, picosulfuric acid (DB09268) is a contact laxative used for constipation treatment or to prepare the large bowel before colonoscopy or surgery. As of this writing, there are no matching documents in PubMed which cite both fluindione and picosulfuric acid. However, it is well known that contact laxatives might significantly reduce absorption of orally administered drugs. Specifically, using fluindione and picosulfuric acid concomitantly, the absorption of fluindione into the systemic circulation may be reduced, lower concentrations of fluindione at its binding site may be expected and consequently, fluindione (which acts systemically—not locally) would be less effective.

Mefloquine (DB00358) is a phospholipid-interacting antimalarial drug, very effective against Plasmodium falciparum and with relatively few side effects. Orlistat (DB01083) on the other hand is a drug designed to treat obesity. Its primary function is preventing the absorption of fats from the human diet by inhibiting pancreatic lipases, enzymes that break down triglycerides in the intestine. There are no documents in the PubMed which match both mefloquine and orlistat. However, we may anticipate the pharmacokinetic interaction between both the compounds at the level of absorption. Mefloquine is a rather fat-soluble molecule and its absorption could be significantly decreased while taking orlistat concomitantly.

Phenobarbital (DB01174) is a barbituric acid derivative that acts as a nonselective central nervous system depressant. It promotes binding to inhibitory gamma-aminobutyric acid subtype receptors, and modulates chloride currents through receptor channels. Atomoxetine (DB00289) on the other site is the first non-stimulant drug approved for the treatment of attention-deficit hyperactivity disorder (ADHD). In PubMed database there are only two documents referring to both compounds. Phenobarbital is a strong inducer of the liver enzymes of the cytochrome P450 system, through which many drugs are metabolized. Biotransformation of atomoxetine in human is predominantly carried out with isoenzyme CYP2D6. The isoenzyme is induced by concomitant administration of phenobarbital. Atomoxetine is thus rapidly metabolized to its inactive metabolites. Consequently, plasma concentrations of atomoxetine are decreasing rapidly with time which could result in lower atomoxetine therapeutic effect.

Ouabain (DB01092) is a cardiotonic glycoside obtained from the seeds of Strophanthus gratus and other plants and has a long history in the treatment of heart failure, angina pectoris and myocardial infarction. The drug acts as an inhibitor of the Na^+^/K^+^-enhancing ATPase. Digitoxin (DB01396) is also a cardiac glycoside, phytosteroid, similar in structure, mechanism of action and effects to digoxin (Na^+^/K^+^-enhancing ATPase), although digitoxin effects are longer-lasting. There are 391 documents in PubMed which cite both ouabain and digitoxin, although manual review of the abstracts reveals no paper in which both ouabain and digitoxin are described as interacting compounds. If both compounds would be administered concomitantly without adjustment of the dosing regimen, the pharmacological effects were expected to be excessive (e.g., cardiac arrest, atrioventricular block), especially for the reason that both drugs have a very narrow therapeutic window.

## Discussion

In this study we performed a computational approach to potential DDIs identification using computer-based link prediction techniques. We performed link prediction on the five arbitrary chosen large-scale DDI networks from bioinformatics domain. The main conclusion of this research is that supervised link prediction demonstrated to be a plausible methodology for DDIs prediction. The predicted power is very high for all major DDI databases. In addition, results of this study clearly show that topological information is more important in predicting novel DDIs than semantic information. Our approach is especially useful for large-scale DDI networks for detection of potential associations among drugs whose biological roots are not completely explained.

There is a huge interest to understand and elucidate DDIs in contemporary science. As in other life science issues, network-based pharmacology offers a convenient back-up to mechanistic and molecular modeling [57]. Due to the high cost of experimental data and therefore lack of empirical evidence, the use of computerized machinery to predict DDIs has been highly encouraged [33]. Of course, analysis of potential interactions could lead to interesting discoveries, but cannot substitute the actual pharmacological introspection. It would be necessary in the future to include also genomic covariates and free-text data. We limit our analysis only to selected, large-scale databases and do not include other, perhaps clinically more relevant databases (e.g., Drugs.com, Medscape Multi-Drug Interaction Checker, RxList). However, the main limitation of the aforementioned databases is that they do not offer a public application programming interface (API) or downloadable database that would greatly expand possibilities for massive data mining. Next, our analyis is based only on potential interactions which represent the simplest way of relationships between drugs. Potential interactions can be treated as pure co-occurrences and not as meaningful links among drugs. This imperfection could be probably circumvented by the introduction of semantic relations. Using semantic relations approach, the relationships between drugs can be described with greater expressiveness and much more accurately. Finally, we do not consider weights on links and treat all interactions as equal. We may expect that the weighting scheme would greatly improve prediction preformance of the presented approach. Especially in bio-inspired networks the confidence score (i.e., edge weight) is as important as presence of relationship [58].

Despite the large number of approaches, link prediction in large-scale networks is still a very challenging problem. There are also many possibilities for further work. First and most important, we should dig into the problem of cleaning (i.e., filtering) interactions in the network. A small portion of detected interactions in the analysed networks may be also excessive. In this regard it is crucial to distinguish between potential and actual, clinically confirmed interactions among drugs. Describing the practical importance of an interaction is essential due to a myriad of potential, but clinically not significant, associations [59]. Currently, there are no special protocols for determining if particular interaction is clinically relevant [60]. To address this issue, we will use UMLS filtering as we suggested elsewhere [61]. Using UMLS Metathesaurus each drug may be linked to a semantic type. Next, the UMLS Semantic Network then provides a set of allowable links between these drugs. We strongly believe that such filtering will greatly increase validity of the proposed methodology. Second, our approach only considers static network snapshots. However, DDIs network is a dynamic system that could expand in terms of size and space over time. Hence, the classification task could be augmented to incorporate dynamic aspects of an evolving network. In addition, our intention is to develop a Web-based application that will exploit presented approach for DDI network curation and make it available to broad community of researchers. Last but not least, it is also straightforward to extent this work to drug-target, drug-disease or drug-food interactions.

## Conclusion

Link prediction is a promising methodological framework for studying complex systems in different scientific disciplines, including pharmacology. We evaluate an approach to potential DDIs prediction using link prediction methodology. We study the prediction performance of unsupervised and supervised link prediction algorithms on several large-scale DDI networks. Although there exist many different approaches and algorithms, reliable prediction of links in a network is still a very challenging problem. Computational approach presented here can be used as tool to help researchers to identify potential DDIs. Overall, our results demonstrated favorable classification performance and suggest appropriateness of the presented methodology for potential DDIs identification.

## Supporting information

S1 Table Classification rates for unsupervised link prediction on training and test data. (PDF)

S2 Table Classification rates for supervised link prediction on training data. (PDF)

S3 Table Classification rates for supervised link prediction on test data. (PDF)

S4 Table Feature importance. Absolute and relative average feature importance for all learning models for all included networks.
(PDF)
