# Supplementary material for: Predicting potential drug-drug interactions on topological and semantic similarity features using statistical learning
Source: PLoS One. 2018 May 8;13(5):e0196865. doi: 10.1371/journal.pone.0196865 (PMC5940181; doi:10.1371/journal.pone.0196865)
Supplement: S3 Table — (PDF) [file pone.0196865.s003.pdf]

Predicting potential drug-drug interactions on topological and semantic similarity features using statistical learning

Supplementary information

Andrej Kastrin Polonca Ferk Brane Leskošek

Table S3: **Classification performances for link prediction pipeline on test data.**

Network	Classifier	$\mathbf{FN_{rate}}$	$\mathbf{FP_{rate}}$	$\mathbf{TN_{rate}}$	$\mathbf{TP_{rate}}$
DrugBank	DT	0.01	0.45	0.55	0.99
	kNN	0.01	0.34	0.66	0.99
	SVM	0.01	0.42	0.58	0.99
	RF	0.01	0.21	0.79	0.99
	GBM	0.01	0.35	0.65	0.99
KEGG	DT	0.01	0.68	0.32	0.99
	kNN	0.01	0.65	0.35	0.99
	SVM	0.00	0.79	0.21	1.00
	RF	0.01	0.55	0.45	0.99
	GBM	0.01	0.63	0.37	0.99
NDF-RF	DT	0.00	0.88	0.12	1.00
	kNN	0.00	0.97	0.03	1.00
	SVM	0.00	0.93	0.07	1.00
	RF	0.00	0.90	0.10	1.00
	GBM	0.00	0.85	0.15	1.00
SemMedDB	DT	0.00	0.75	0.25	1.00
	kNN	0.00	0.70	0.30	1.00
	SVM	0.00	0.71	0.29	1.00
	RF	0.00	0.68	0.32	1.00
	GBM	0.00	0.69	0.31	1.00
Twosides	DT	0.08	0.18	0.82	0.92
	kNN	0.07	0.23	0.77	0.93
	SVM	0.06	0.20	0.80	0.94
	RF	0.06	0.18	0.82	0.94
	GBM	0.06	0.17	0.83	0.94

Legend: FN_{rate} – false negative rate, FP_{rate} – false positive rate, TN_{rate} – true negative rate, TP_{rate} – true positive rate.

Four measures presented in Table S3 measure the classification performance on positive and negative classes independently:

False negative rate: $FN_{rate} = FN/(TP + FN)$ is the proportion of positive cases misclassified as belonging to the negative class;

False positive rate: $FP_{rate} = FP/(FP + TN)$ is the proportion of negative cases misclassified as belonging to the positive class;

True negative rate: $TN_{rate} = TN/(FP + TN)$ is the proportion of negative cases correctly classified as belonging to the negative class;

True positive rate: $TP_{rate} = TP/(TP + FN)$ is the proportion of positive cases correctly classified as belonging to the positive class.

The meaning of abbreviations is given in Figure 1.

		Reference	
		Positive	Negative
Prediction	Positive	True Positive (TP)	False Positive (FP)
	Negative	False Negative (FN)	True Negative (TN)

Figure 1: Confusion matrix
