# Supplementary material for: Predicting potential drug-drug interactions on topological and semantic similarity features using statistical learning
Source: PLoS One. 2018 May 8;13(5):e0196865. doi: 10.1371/journal.pone.0196865 (PMC5940181; doi:10.1371/journal.pone.0196865)
Supplement: S4 Table — Absolute and relative average feature importance for all learning models for all included networks. (PDF) [file pone.0196865.s004.pdf]

Predicting potential drug-drug interactions on topological and semantic similarity features using statistical learning

Supplementary information

Andrej Kastrin Polonca Ferk Brane Leskošek

Table 1: **Feature importance.** Absolute and relative average feature importance for all learning models for all included networks. For each model we quantified relative importance by a weight between 0 and 1 for each feature.

		RF		GBM	
**Network**	**Feature**	**Abs**	**Rel**	**Abs**	**Rel**
DrugBank	CN	5982.41	0.84	411.80	0.02
	RAI	3310.43	0.44	18599.92	1.00
	JC	3981.78	0.54	739.43	0.04
	AAI	6531.66	0.92	556.97	0.03
	PA	2461.18	0.31	377.74	0.02
	CCN	7086.17	1.00	4142.33	0.22
	CRA	1520.46	0.17	668.48	0.04
	WIC	968.03	0.09	340.88	0.02
	ATC	393.99	0.00	8.70	0.00
	CHEM	1312.08	0.14	0.66	0.00
	MESH	1410.42	0.15	69.36	0.00
	ADE	687.81	0.04	0.00	0.00
KEGG	CN	315.80	0.48	68.76	0.09
	RAI	493.41	0.83	256.36	0.37
	JC	426.31	0.70	128.45	0.18
	AAI	466.09	0.78	49.76	0.07
	PA	577.87	1.00	574.68	0.83
	CCN	398.66	0.65	693.28	1.00
	CRA	306.32	0.46	287.46	0.41
	WIC	213.31	0.28	78.63	0.11
	ATC	72.30	0.00	3.48	0.00
	CHEM	276.14	0.40	33.80	0.04
	MESH	302.67	0.46	43.84	0.06
	ADE	134.70	0.12	11.36	0.01
NDF-RT	CN	315.80	0.05	68.76	0.05
	RAI	493.41	0.34	256.36	0.18
	JC	426.31	0.39	128.45	0.25

Continue on the next page

Table 1: **Feature importance (cont'd).**

Network	Feature	RF Abs	RF Rel	GBM Abs	GBM Rel
	AAI	466.09	0.32	49.76	0.15
	PA	577.87	1.00	574.68	1.00
	CCN	398.66	0.11	693.28	0.00
	CRA	306.32	0.16	287.46	0.25
	WIC	213.31	0.10	78.63	0.21
	ATC	72.30	0.00	3.48	0.01
	CHEM	276.14	0.41	33.80	0.05
	MESH	302.67	0.50	43.84	0.05
	ADE	134.70	0.20	11.36	0.06
SemMedDB	CN	328.49	0.70	7.42	0.00
	RAI	427.93	1.00	833.51	1.00
	JC	308.19	0.64	43.62	0.04
	AAI	399.00	0.91	61.35	0.07
	PA	309.89	0.65	63.18	0.07
	CCN	326.16	0.70	101.33	0.11
	CRA	252.22	0.47	78.17	0.09
	WIC	105.00	0.03	22.38	0.02
	ATC	93.56	0.00	9.99	0.00
	CHEM	206.73	0.34	21.55	0.02
	MESH	258.06	0.49	147.06	0.17
	ADE	133.22	0.12	9.82	0.00
Twosides	CN	219.70	0.55	9.59	0.00
	RAI	195.03	0.49	65.15	0.04
	JC	125.20	0.29	108.49	0.07
	AAI	223.19	0.56	21.04	0.01
	PA	378.23	1.00	1402.98	1.00
	CCN	161.85	0.39	11.41	0.00
	CRA	34.26	0.03	34.12	0.02
	WIC	22.45	0.00	17.71	0.01
	ATC	30.44	0.02	25.90	0.01
	CHEM	47.22	0.07	9.73	0.00
	MESH	47.94	0.07	25.31	0.01
	ADE	46.40	0.07	18.34	0.01

Legend: RF – random forest, GBM – gradient boosting machine, Abs – absolute feature importance, Rel – relative feature importance
